# Metropolitan Hospitals Ancient and Modern

**Published:** 1889-01-19

**Authors:** 


### Metropolitan Hospitals Ancient and Modern.

(By a Medical Superintendent.)

*( Concluded from page 240. )*

### IV.—OUT AND IN PATIENTS AND THE PAY SYSTEM.

But however strong our desire to see the hospitals fully occupied, it is not less incumbent that some check should be placed on that thriftless improvidence which considers it has a prescriptive right to gratuitous medical relief because the charity is supported at public expense. In this matter the hospitals themselves, especially the older ones, are not altogether free from blame. For a hundred years and more their doors have been open to all who chose to take advantage of their good offices, and it is hardly surprising that the indiscriminate relief afforded should have sapped the springs of those principles of self-respect and independence which are being continually inculcated on the multitude from the pulpit and the press, and by all wise philanthropists. A few years ago the demoralising effect, especially on out-patients, was a fertile theme for discussion, and numerous remedies have from time to time been suggested to control, if it is impossible to avert, the evil. Charity boxes for thankofferings are freely displayed in most hospitals, but the sum of their contents is conspicuously small, while in some cases an officer is appointed to inquire into the means and substance of the few, who by reason of the decent character of their external habiliments may not be thought worthy of gratuitous relief. To be successful the test and responsibility must rest with the patient and not with the hospital authorities, and the only plan hitherto suggested which has been at all effective is the imposition of a small payment, not sufficient to cover the cost of the drugs, but enough to inculcate the wholesome lesson that physic costs money, whatever value the patient may place on the advice. There is no rule without exception, and the exceptions become very numerous indeed in dealing with medical charity, which necessitates every precaution lest the poor should suffer on account of their poverty. It is found by the experience of the numerous establishments into which the pay system, as it is called, has been introduced, that very few, not more than two per cent. it is said, express their inability to pay. It is but fair that such should receive gratuitous assistance in the first instance, but that they should fortify their statement on their next visit with a certificate from a clergyman or from a respectable householder acquainted with their capabilities.

With respect to the patients received into the hospitals the case is very different. Nine-tenths of them are unable to pay anything towards their maintenance; but the remainder are able to pay, either themselves or through their employers, from 5*s.* to 20*s.* a-week towards their maintenance, as is proved by the experience of numerous institutions. The increment derived from hospital patients shows, from the statistics already quoted, either that the self-help principle is developing among the masses, or that the hospitals are possibly being taken advantage of by a class for whom they were scarcely intended in the first instance, but who require their help just as much as the others. It has always been an opprobrium of the London and large provincial hospitals that strangers suffering from accident or illness and willing to pay towards their treatment could not be received into any but the ordinary beds unless they were recommended by a subscriber, or their complaints were of too severe a character to justify their rejection. Mr. Burdett, in his book on pay hospitals, has shown how foreign nations were ahead of us in this respect,—how private wards and separate apartments were attached to most hospitals on the Continent and in America, while no such privileges were enjoyed by the middle classes at home. The question with respect to infectious disease was finally set at rest by the Small Pox and Fever Hospitals, both charitable foundations, charging admission fees from their patients at so much per week when admitted into private apartments, and a less sum for treatment in the general wards. By this departure many have been saved the invidious distinction of becoming paupers who would otherwise have been forced to take refuge in one or other of the infectious hospitals of the Sick Asylums board. The difficulty of applying a like principle to ordinary cases of medical and surgical disease is naturally much greater in old institutions than in many modern charities, whose very existence depends in a measure on the payments made by the patients. This principle is mostly in vogue in hospitals formed for the treatment of consumption, in special hospitals for women, for children, for diseases of the throat, and in the numerous cottage hospitals which have sprung up of late years in various parts of the country. A more earnest, if not more ambitious attempt to meet what everyone considered to be a great want, was made some years ago by the establishment of a veritable pay hospital on Wandsworth Common.

If we are rightly informed, this was intended for a class of people both above, and on a par with ordinary hospital patients, who were required to pay from 10*s.* to £3 3*s.* a week according to the accomodation provided. The house appears to have been fairly taken advantage of; but possibly from its distance from the metropolitan centre and its not being incorporated with the clientèle of a medical school, it has not met with the success it justly merits. The pay hospital, which Mr. Burdett was instrumental in organising in Fitzroy Square, hardly meets the requirements of the class to whom we refer, but, on the other hand, it must be hailed as a boon to surgeons and patients alike, on account of its excellent nursing and sanitary arrangements. Although lacking some of the advantages of Fitzroy House, private institutions of a kindred character have been established in the West End of London, generally through the enterprise of experienced nurses, and must prove a great convenience to medical men, as well as patients. But we will now consider what has been doing in the older hospitals, not only to meet the want referred to, but as a legitimate source of gain to the institutions themselves. The case of the special hospitals has been already referred to as initiating the principle of self-help, but an attempt on a large scale was made some ten years since by the governors of St. Thomas's Hospital, who felt anxious to utilise a portion of their vacant wards for middle-class purposes. A couple of large wards were consequently split up into compartments or cubicles for the accommodation of forty patients, who could afford to pay about £3 3*s*. a-week for their maintenance, medical attendance, and nursing. A competent medical officer was also specially appointed to take sole charge of the patients, who, however, had it in their power to call in the assistance of any medical man from the outside if they cared to pay for it. It had been estimated by the authorities—and not without reason—that the payments from the patients, provided the cubicles were kept moderately well occupied, would not only cover all expenses, but realise a considerable surplus for the hospital benefit. The experiment has now been in operation for several years, and, after allowing for initial expenses and tear and wear, we are informed that the average gains amount to £1,000 a-year, a sum sufficient to show that the privilege has been duly appreciated by a class whose needs have hitherto received but little attention. Simultaneously with this innovation facilities were afforded to people for admission to the general wards, who of themselves or through their friends or employers were able to contribute £1 1*s*. per week towards their expenses. For this sum they were to receive all the privileges which a hospital affords, that is to say, attendance by the visiting and resident medical staff, nursing, etc., but in these and other respects they were no better off than the bulk of patients occupying beds in the same wards who were fortunate enough to pass the admission ordeal without any demur as to their social eligibility. Somewhat similar arrangements were afterwards carried into effect at Guy's Hospital, and have been attended with equally beneficial results, the bulk of the patients availing themselves of the privileges having in the first instance been induced to do so by their own medical attendants.

It is becoming more evident day by day that if charity is to be judiciously and not vicariously dispensed it is essential that the recipients should be made to feel that the benefits bestowed on them with no ungenerous hand are worthy of something more than empty thanks. Each and all are bound to do what in them lies for the ready help afforded. Considering also that there are many of the lower middle-class desirous of participating in hospital privileges, and are ready to pay a reasonable sum for them, it would appear incumbent on all hospitals that they should make such a department an essential part of their economy.

No doubt there must always exist some difference of opinion as to who are and who are not fitting subjects for hospital relief, and the expediency of some of the suggestions offered in the context may be open to question, but there is sure to be a consensus of opinion as regards the necessity of a well-ordered economy in the administration of charitable trusts. We hear this sentiment so constantly reiterated in connexion with the medical charities of London that a stranger might be pardoned the inference that they were the cheapest conducted establishments in the world, but in the face of a growing expenditure out of proportion with the numbers relieved and largely in excess of what obtains in other establishments of a kindred character, provincial and foreign, it is high time that the maxim should undergo a stricter interpretation. People who contribute to hospitals have a right to know that their money is laid out to the utmost advantage, and for the benefit of the greatest number, consistent with efficiency of administration. We are not less keenly alive to the difficulties and almost helplessness of the executive authorities in dealing with evils, which once introduced into the hospital system, cling like a canker-worm and render nugatory the best resolves. Still, though the struggle be a hard one, much may be done in curtailing waste by a closer attention being given to that multitude of minor details incorporated with the well-being and economy of every hospital, which, after all that has been said, constitutes the main source of its expenditure.

